# Hormone receptor profile of ectopic and eutopic endometrium in adenomyosis: a systematic review

**DOI:** 10.1093/hropen/hoaf002

**Published:** 2025-01-20

**Authors:** Alison Maclean, Laura Tipple, Emily Newton, Dharani K Hapangama

**Affiliations:** Department of Women’s and Children’s Health, Institute of Life Course and Medical Sciences, University of Liverpool, Liverpool, UK; Centre for Women’s Health Research, Liverpool Women’s Hospital, Liverpool, UK; School of Medicine, University of Liverpool, Liverpool, UK; The Hewitt Fertility Centre, Liverpool Women’s NHS Foundation Trust, Knutsford, UK; Department of Women’s and Children’s Health, Institute of Life Course and Medical Sciences, University of Liverpool, Liverpool, UK; Centre for Women’s Health Research, Liverpool Women’s Hospital, Liverpool, UK

**Keywords:** adenomyosis, androgen receptor (AR), ectopic endometrium, eutopic endometrium, G protein-coupled oestrogen receptor (GPER), gonadotrophin-releasing hormone receptor (GnRH-R), hormone receptors, oestrogen receptor (ER), progesterone receptor (PR), systematic review

## Abstract

**STUDY QUESTION:**

What is the hormone receptor profile of adenomyosis lesions in comparison to correctly located endometrium?

**SUMMARY ANSWER:**

Adenomyosis lesions exhibit increased oestrogen receptor (ER) expression compared to the eutopic endometrium; there are conflicting results regarding progesterone receptor (PR) expression and a lack of studies on androgen receptor (AR) expression.

**WHAT IS KNOWN ALREADY:**

Adenomyosis lesions express hormone receptors indicating an influence from ovarian steroid hormones. However, hormone treatments are often ineffective in controlling adenomyosis symptoms, which suggests alternate hormonal responses and, potentially, a distinct hormone receptor expression profile within adenomyosis lesions compared to the eutopic endometrium.

**STUDY DESIGN, SIZE, DURATION:**

This systematic review with a thematic analysis retrieved studies from the PubMed, Ovid Medline, Embase, Scopus, and Cochrane Library databases, and searches were conducted from inception through to May 2024. Human studies were included and identified using a combination of exploded MeSH terms (‘adenomyosis’) and free-text search terms (‘oestrogen receptor’, ‘progesterone receptor’, ‘androgen receptor’, ‘hormone receptor’).

**PARTICIPANTS/MATERIALS, SETTING, METHODS:**

This review was reported in accordance with the PRISMA guidelines. All studies reporting original data concerning hormone receptors in adenomyosis lesions compared to eutopic endometrium in adenomyosis were included. Studies that did not report original data or provide a review of the field were excluded. Bias analysis was completed for each study using the Newcastle–Ottawa scoring system.

**MAIN RESULTS AND THE ROLE OF CHANCE:**

There were 1905 studies identified, which were screened to include 12 studies that met the eligibility criteria, including 11 proteomic studies and one transcriptional study, with a total of 555 individual participants. ER expression was consistently increased in adenomyosis lesions compared to the eutopic endometrium, specifically in the secretory phase. When endometrial subregion was considered, this difference was specific to the endometrial functionalis only. When different isoforms were considered, this increase in ER expression was specific to ERα rather than ERβ. There were conflicting results on PR expression, with most studies showing no significant difference or reduced levels in adenomyosis lesions compared to the eutopic endometrium. There is a paucity of data on AR expression in adenomyosis lesions, with only one study of small sample size included.

**LIMITATIONS, REASONS FOR CAUTION:**

A high risk of bias arose from studies grouping endometrial samples across different menstrual cycle phases for analysis. The coexistence of gynecological conditions like endometriosis may also confound the hormone receptor profile of the eutopic endometrium. Most studies employing immunostaining did not comment on region-specific differences in the endometrium. Given the well-documented cyclical variations in hormone receptor expression within the endometrium, the need for more attention to region-specific differences represents a notable limitation in the current body of literature.

**WIDER IMPLICATIONS OF THE FINDINGS:**

The systematic review highlights oestrogen dominance through elevated ERα levels in adenomyosis lesions, which agrees with the literature suggesting local hyper-oestrogenism in adenomyosis lesions. Heterogeneity in menstrual cycle timing and lack of endometrial region specificity prevent conclusions on progesterone resistance within adenomyosis lesions in this study. Future investigations should minimize the bias through well-defined cohorts, leading to robust exploration of hormone receptor profiles in adenomyosis lesions to identify therapeutic targets and deepen our understanding of adenomyosis pathogenesis.

**STUDY FUNDING/COMPETING INTEREST(S):**

This work was supported by Wellbeing of Women Research Project grants RG1073 and RG2137 (D.K.H.), a Wellbeing of Women Entry-Level Scholarship ELS706 and a Medical Research Council grant MR/V007238/1 (A.M. and D.K.H.), as well as the University of Liverpool (L.T.). There are no conflicts of interest.

**HROPEN-24-0294.R2:**

The review protocol was published in the PROSPERO Register of Systematic Reviews on 27 September 2023, registration number CRD4202346.

WHAT DOES THIS MEAN FOR PATIENTS? Adenomyosis is a gynaecological condition in which the inner lining of the womb, the endometrium, grows abnormally into the muscle layer of the womb, the myometrium. The normal endometrium changes under the influence of hormones such as oestrogen and progesterone, which act through hormone receptors. Adenomyosis lesions also have hormone receptors, but it is not known whether these lesions respond to hormones in the same way as the correctly located endometrium. Hormone treatments are often used to treat adenomyosis but with varying degrees of effectiveness. It is possible that this is because the lesions have a different hormone receptor profile, and understanding this further may lead to better understanding of how to treat the condition.

This review has gathered all the current research looking at the hormone receptors present in adenomyosis lesions compared with the normally located endometrium. The findings have shown that within adenomyosis lesions there are more oestrogen receptors, suggesting that they are exposed to more oestrogen than the normally located endometrium. Previous studies have suggested that adenomyosis endometrium may be resistant to progesterone. If the endometrium is resistant to progesterone, it would be expected that there would be fewer progesterone receptors in the first part of the menstrual cycle, i.e. the proliferative phase, and more progesterone receptors in the second part of the menstrual cycle, i.e. the secretory phase. The studies included in this review often grouped samples from different menstrual cycle phases, making it difficult to conclude whether there is progesterone resistance in adenomyosis lesions compared to the endometrium. There are very few studies looking into androgen receptors in adenomyosis. Future studies are needed to confirm whether the adenomyosis lesions have a different response to progesterone and androgens than the normally located endometrium. This review suggests that future studies consider the menstrual cycle phase and the different regions of the endometrium when studying the hormone receptor profile of adenomyosis lesions and endometrium from women with adenomyosis. A thorough understanding of this will lead to better understanding of why hormone treatments are not effective in many women with adenomyosis and contribute towards developing more effective treatments in the future.

## Introduction

Adenomyosis is defined by the ectopic location of endometrial-like tissue in the myometrium of the uterus. It is considered a hormone-responsive condition since the disease process and associated symptoms are presumed to settle after menopause with the cessation of ovarian hormone production (Leyendecker and Wildt, 2011; Vannuccini *et al.*, 2017; Maclean *et al.*, 2023). The literature on hormone receptor expression in adenomyosis endometrium is growing, and ovarian steroid hormones have been implicated in adenomyosis, with local rather than systemic hormone regulation being involved in the pathogenesis (Kitawaki *et al.*, 2001; Rizner, 2016). Classical hormone regulation of endometrium and endometrium-like tissue is exerted through hormones interacting with their cognate receptors; thus, changes in hormone receptor levels in a tissue are expected to mirror the related hormone responsiveness. The three main ovarian hormones regulating the human endometrium are oestrogens, progesterones, and androgens (Hapangama *et al.*, 2015; Kamal *et al.*, 2016).

**Figure 1.**
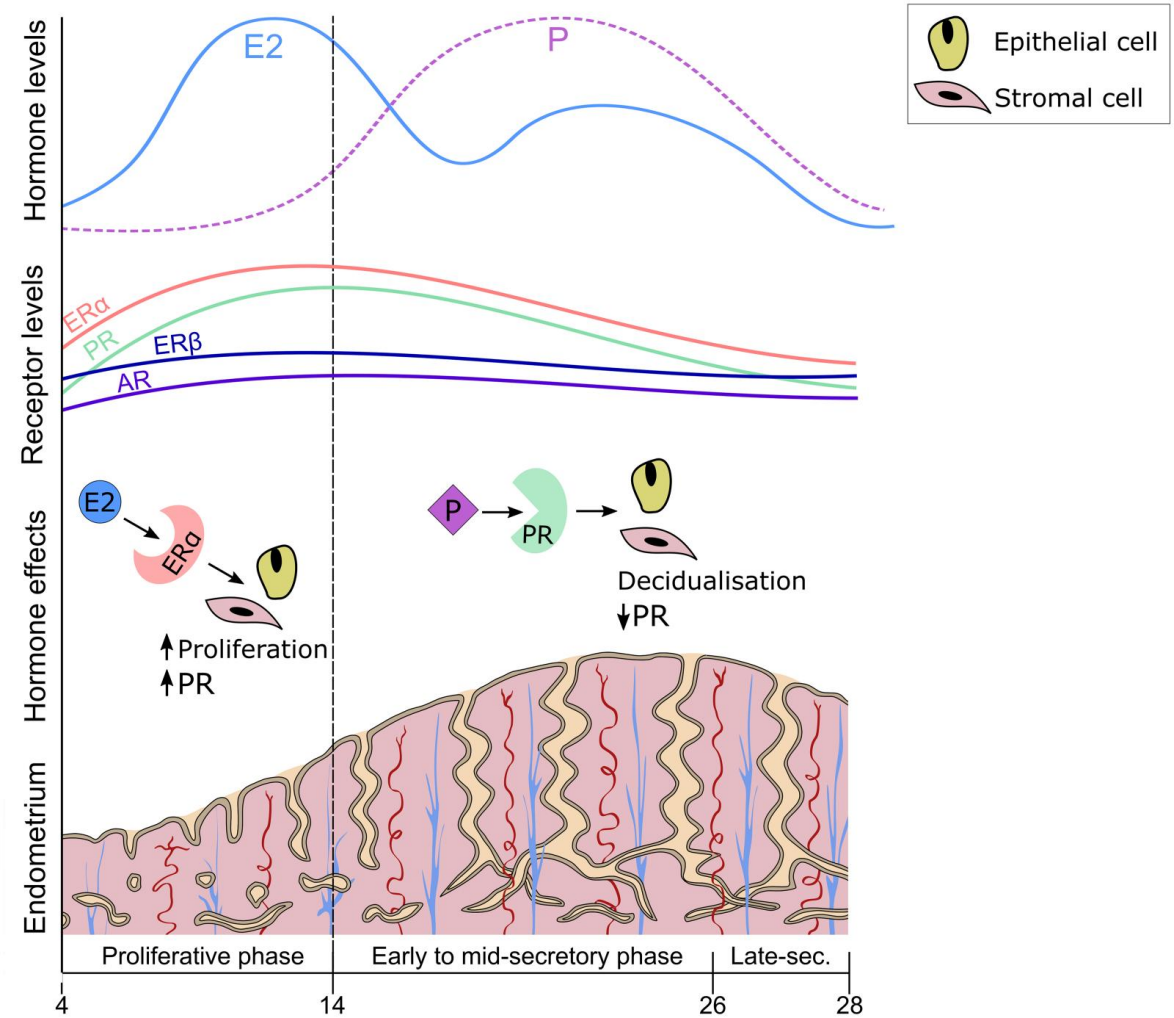
**Schematic illustration of the fluctuations in hormone levels and receptor expression throughout the proliferative and secretory phases of the menstrual cycle.** Serum levels of oestradiol (E2, solid blue line) and progesterone (P, dashed purple line) are shown across the cycle. Expression patterns of oestrogen receptor alpha (ERα, orange), oestrogen receptor beta (ERβ, navy blue), progesterone receptor (PR, green), and androgen receptor (AR, purple) in the endometrial functionalis. Key cellular effects of E2 binding to ERα during the proliferative phase, including increased cell proliferation. Consequences of P binding to PR in the secretory phase, such as decidualization. Arrows indicate increase (↑) or decrease (↓) in receptor expression or cellular processes. The transition from the proliferative to the secretory phase is marked by ovulation (dotted line). This schematic is a simplified representation and does not reflect absolute quantitative values. Receptor expression patterns vary between different cell types and regions within the endometrium.

The endometrial region-specific and cyclical variation in hormone receptor expression is well documented. Oestrogen receptors alpha (ERα) and beta (ERβ), encoded by *ESR1* and *ESR2* genes, respectively, are key targets of oestrogens (E2) in the endometrium (Hapangama *et al.*, 2015). In the proliferative phase (PP) of the menstrual cycle, E2 acting via ERα causes epithelial and stromal cell proliferation and then upregulates progesterone receptor (PR) to prepare for potential embryo implantation (see Fig. 1; Parkes *et al.*, 2018; Yu *et al.*, 2022). The expression of ERα is evident in both epithelial and stromal cells of the endometrial functionalis and basalis layers in the PP, with increased expression in the functionalis compared to the basalis in the late PP. After ovulation, in the secretory phase (SP), progesterone causes downregulation of ERα and PR, opposing the mitogenic effects of E2 and triggering endometrial stromal cell decidualization (Jabbour *et al.*, 2006; Critchley *et al.*, 2020). This is the hallmark of a receptive endometrium, which is essential for embryo implantation (Hsueh *et al.*, 1975; Katzenellenbogen, 1980; Hapangama, 2003; Hapangama *et al.*, 2015). The SP endometrium is characterized by lower ERα and PR expression in the functionalis glands and, to a lesser extent, stroma (see Fig. 1; Snijders *et al.*, 1992; Critchley *et al.*, 2001). The basalis endometrium exhibits less cyclical variation in ERα and PR despite exposure to the same circulating steroid hormones, indicating that it is less hormonally responsive than the functionalis (Lecce *et al.*, 2001; Mylonas *et al.*, 2004; Critchley and Saunders, 2009; Kamal *et al.*, 2016). ERβ, which is expressed in all cell types in the human uterus, plays a role in preventing undesired ERα-mediated actions of E2; this is a key regulatory mechanism in the endometrium (Hapangama *et al.*, 2015). ERβ is expressed in the functionalis and basalis layers of the human endometrium and exhibits less cyclical variation than ERα (see Fig. 1). The G protein-coupled oestrogen receptor (GPER) may also be involved in the E2-driven physiological changes in the human endometrium. Endometrial GPER expression varies across the menstrual cycle, with increased expression in the E2-dominant PP (Plante *et al.*, 2012) and the lowest expression observed during the SP (Zimmerman *et al.*, 2016), a pattern similar to ERα.

Local hyper-oestrogenism in adenomyosis has been suggested by the finding of elevated levels of E2 in the menstrual blood of women with adenomyosis compared to those without (Takahashi *et al.*, 1989) and elevated levels in menstrual blood compared with peripheral blood (Rizner, 2016). Polymorphisms of the ERα gene are associated with an increased risk of adenomyosis through increased receptor activity (Kitawaki *et al.*, 2001; Oehler *et al.*, 2004), and the expression levels of ERα and ERβ in the eutopic endometrium of women with adenomyosis have been found to be significantly higher compared to women without the condition (Sztachelska *et al.*, 2022). The prevailing theory of adenomyosis lesion formation through the invagination of endometrial basalis cells into the myometrium involves E2-induced epithelial to mesenchymal transition, whereby endometrial epithelial cells lose their cell–cell adhesion properties and acquire mesenchymal characteristics with enhanced migratory ability. This transformation allows them to invade the myometrium and form adenomyosis lesions (Sigurgeirsson *et al.*, 2017; Chen *et al.*, 2024). The tissue injury and repair theory (TIAR) of adenomyosis pathogenesis is further linked with the concept of hyper-oestrogenism in adenomyosis. It is proposed that increased local release of E2 due to paracrine activity in the endometrium of women with adenomyosis may enhance uterine contractility. This creates a vicious cycle of a local hyper-oestrogenic milieu, characterized by increased local release of E2, auto-traumatization, and wound healing, which promotes inflammation and further production of local E2 (Garcia-Solares *et al.*, 2018; Rossi *et al.*, 2022). The TIAR theory postulates that injury at the endomyometrial interface could be triggered by physiological events, such as uterine peristalsis during menstruation (Leyendecker and Wildt, 2011). Repetitive menstrual cycles may thus cause cumulative tissue injury over time, leading to a chronic state of repair and inflammation at the endomyometrial junction (Bulun *et al.*, 2023).

Progesterone resistance has been associated with adenomyosis (Vannuccini *et al.*, 2017) and is defined as a decreased responsiveness of the tissue to the effects of progesterone. This results in an inability of progesterone to regulate endometrial functions such as decidualization and may be evident by the reduced expression of PRs. However, it is essential to appreciate the complex regulation of PR expression levels by progesterone itself, since ligand-activated PR reduces the expression of the *PR* gene. Furthermore, understanding the cyclical and region-specific variation of PR in healthy endometrium first will allow accurate interpretation of the hormone receptor profile associated with progesterone resistance. There are two isoforms of PR: PR-A and PR-B. PR-A is considered the dominant isoform and the primary mediator of progesterone action, inhibiting PR-B. PR-A is located in the nuclei of endometrial epithelial and stromal cells during the PP, persisting in the stromal compartment of the functionalis, particularly in the perivascular region (Hapangama, 2003). PR-B mediates specific effects of progesterone. The expression patterns of PR-A and PR-B exhibit dynamic changes throughout the menstrual cycle. In the late PP and early SP, when progesterone levels initially rise, PR-A and PR-B are expressed in the glandular epithelium and stroma of the endometrial functionalis layer. PR-A and PR-B are subsequently downregulated by the progestogenic action in the late SP, resulting in reduced expression in the glands of the endometrial functionalis, more so than in the endometrial basalis (see Fig. 1) (Snijders *et al.*, 1992; Critchley *et al.*, 2001; Hapangama, 2003). PR remains in the functionalis stroma in the SP. In the absence of implantation of a fertilized embryo, the demise of the corpus luteum leads to a decline in progesterone levels and shedding of the upper functionalis during menstruation. Both eutopic and ectopic endometrium in adenomyosis exhibit a reduction in progesterone receptor isoform B (PR-B) immunoreactivity (Jichan *et al.*, 2010). This reduction in PR-B, a key mediator of progesterone action, is proposed to be associated with progesterone resistance in adenomyosis. Progesterone resistance has also been related to the KRAS mutations found within adenomyosis (Inoue *et al.*, 2019) as a potential mechanism; however, this mechanistic pathway is still not fully understood. Studies also suggest that progesterone resistance occurs due to increased ER expression in adenomyosis endometrium (Jichan *et al.*, 2010).

Androgen receptor (AR) is present in both ectopic and eutopic endometrium in adenomyosis (Horie *et al.*, 1992). The AR in the human endometrium plays a role in mediating the effects of androgens, such as testosterone and dihydrotestosterone. Upon binding to androgens, the AR translocates into the cell nucleus and acts as a transcription factor (Simental *et al.*, 1991). The effects of AR on the human endometrium are yet to be fully understood. However, a potential role in the regulation of proliferation is evident by increased AR expression in the functionalis stroma in the PP compared to the SP (Mertens *et al.*, 2001; Slayden *et al.*, 2001; Slayden and Brenner, 2004; Critchley and Saunders, 2009; Kamal *et al.*, 2016). Expression of AR in the basalis layer of healthy eutopic endometrium suggests possible involvement in the maintenance and renewal of the endometrium (Marshall *et al.*, 2011; Kamal *et al.*, 2016), and *in vitro* studies have shown androgens have an anti-proliferative effect on endometrial stromal cells (Brenner *et al.*, 2003). However, knowledge about the role of AR in adenomyosis is limited due to the lack of published literature.

The receptors to hormones of the hypothalamic–pituitary–ovarian (HPO) axis and other pituitary hormones have similarities with ovarian hormone receptors, and they may be present in endometrial tissue with a functional relevance. GnRH from the hypothalamus stimulates the pituitary to release LH and FSH, which act on the ovaries to produce oestrogen and progesterone. These ovarian hormones then feedback to the hypothalamus and pituitary, maintaining hormonal balance and regulating the menstrual cycle (Hapangama, 2024). Notably, GnRH receptors have been found in endometrial tissue, suggesting direct effects beyond its role in ovarian stimulation (Maggi *et al.*, 2016). Additionally, hormone receptors such as thyroid-stimulating hormone (TSH) receptors show affinity for ovarian hormones and may be present in the endometrium, indicating complex endocrine interactions in reproductive processes.

The endometrial functionalis and basalis layers exhibit distinct differences in hormone receptor expression over the menstrual cycle, most evident during the SP. Whether adenomyosis lesions exhibit a similar hormone receptor profile to the endometrial basalis or functionalis is not fully understood. However, it may allow insight into the origin or functional similarities of the endometrial cells forming the lesions, providing further evidence for or against the theory of invasion of endometrial cells into the myometrium.

Although considered a hormone-dependent condition, adenomyosis is often refractory to medical hormonal treatments (Etrusco *et al.*, 2023), resulting in many women choosing to undergo surgery to alleviate symptoms. Hormones regulate changes in the correctly located endometrium; therefore, hormone receptor expression in adenomyotic lesions may explain why adenomyosis is refractory to hormonal treatments in some women. At present, targeted endocrinological therapies that specifically act on adenomyotic lesions while sparing the normally located endometrium do not exist but would represent a sought-after advancement in treatment options. The development of such therapies is hindered by an incomplete understanding of the hormonal regulation of cells within adenomyotic lesions compared to their counterparts in the eutopic endometrium. Elucidating these differences, particularly in hormone receptor expression and signalling pathways, is crucial for explaining variable treatment responses and, more importantly, for identifying novel therapeutic targets.

The primary aim of this study was to systematically review the available literature comparing the expression of hormone receptors in adenomyosis lesions to that in eutopic endometrium in women with adenomyosis.

## Methods

This systematic review was conducted in accordance with the Preferred Reporting Items for Systematic Reviews and Meta-Analyses (PRISMA) guidelines (Page *et al.*, 2021). The review protocol was published in the PROSPERO Register of Systematic Reviews on 27 September 2023, registration number CRD4202346.

### Systematic search

A systematic literature search was performed using Medline, PubMed, Scopus, Embase, and Cochrane Central Library databases. All databases were searched from inception to 16 May 2024. The search string was composed of a combination of medical subject headings (MeSH) terms and free-text search terms for the two study components, ‘adenomyosis’ and ‘hormone receptor expression’. The search strategy can be viewed in Supplementary Table S1. All relevant articles, including those in non-English language, were included.

### Eligibility criteria

All human studies reporting original data concerning the expression of hormone receptors in adenomyosis lesions and eutopic endometrium in adenomyosis were included. The hormone receptors included were ER, PR, GPER, AR, and GnRH-R; no publications on LH, FSH, or TSH were identified. Studies were excluded if they did not report original data, e.g. reviews. Case reports and case series were also excluded. All studies conducted in animals were excluded. Furthermore, studies were excluded if participants had received hormone treatments for 3 months before research tissue was obtained and if participants also had a coexisting diagnosis of endometrial malignancy.

### Study selection

The search results were collated, and duplicates were identified and removed. Title and abstract screening to identify studies meeting the eligibility criteria was completed independently by two authors using the online manuscript screening software Rayyan (Ouzzani *et al.*, 2016). Full texts were retrieved and evaluated using the pre-determined eligibility criteria. Any disagreements were resolved through discussion, and a consensus was achieved. Additional studies were identified through forward and backward chaining of the included studies. The references of all the literature and systematic reviews identified by the original search were also screened.

### Data extraction and thematic synthesis

Data extracted from the included studies included the title, authors, journal, year of publication, mode of diagnosis of adenomyosis, menstrual cycle phase at the time of biopsy collection, hormone receptor studied, experimental technique used, primary results, and outcomes. The results were synthesized in a thematic analysis (Supplementary Table S2). The authors identified recurring themes within the included studies. The authors discussed and confirmed the final list of themes.

### Bias analysis

The Newcastle–Ottawa Scale (NOS) was used to assess the quality of each study included in this review (Wells *et al.*, 2013). This scale gives a score out of 9 based on three categories: selection, comparability, and exposure. The categories are allocated a score with a maximum of 4, 2, and 3, respectively. Scores of 0 or 1 in selection exposure highlight an increased risk of bias in these categories, while a score of 0 in the comparability section gives a study a high risk of bias.

## Results

### Study selection

**Figure 2.**
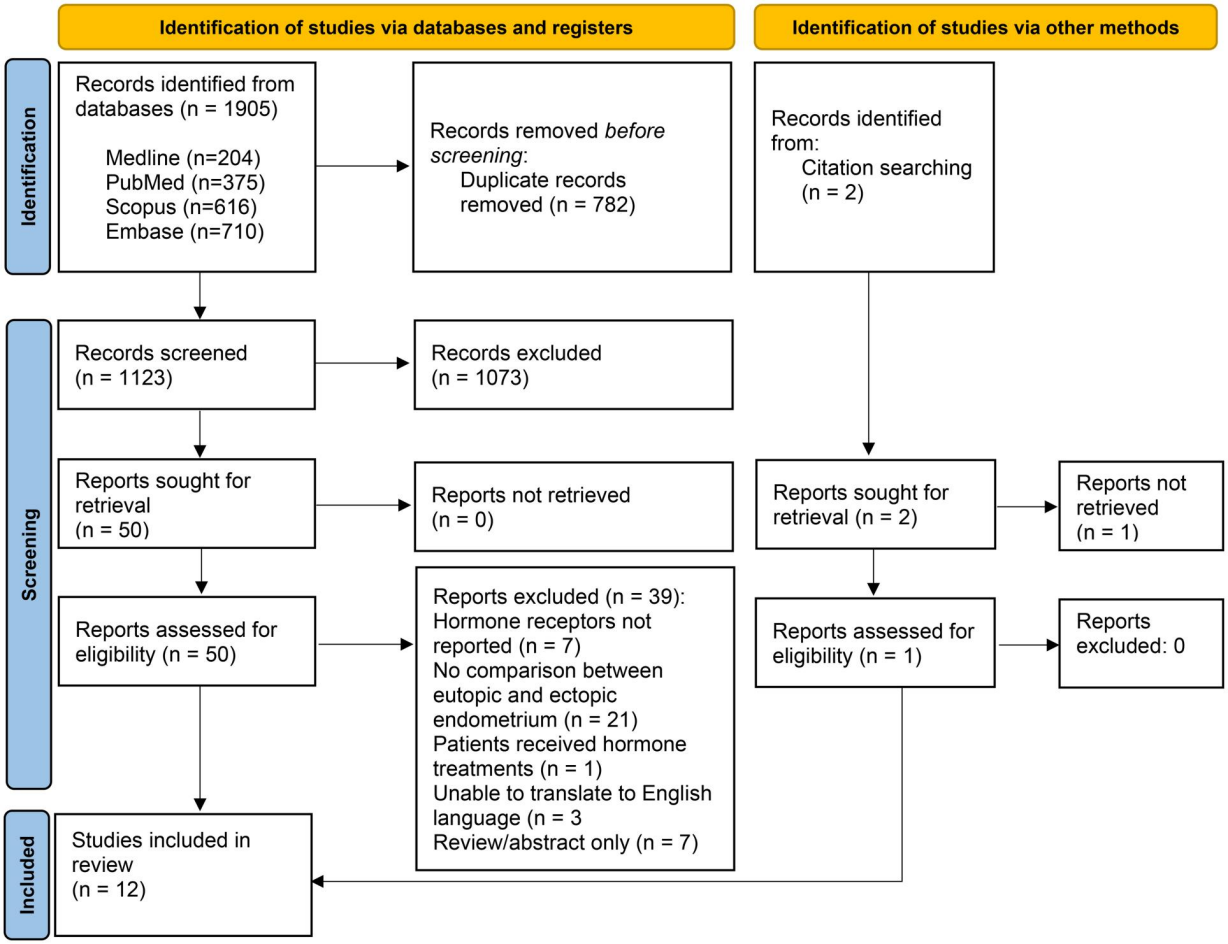
**Preferred Reporting Items for Systematic Reviews and Meta-Analyses (PRISMA) flow diagram illustrating the identification of studies at each stage of the systematic search process and highlighting the number of records included in the final review.** Arrows indicate the progression of the number of studies identified through each stage, with the reason for exclusion where applicable (Page *et al.*, 2021). For more information, visit: http://www.prisma-statement.org/.

The search strategy identified a total of 1905 publications. There were 1123 studies remaining after the removal of duplicates. Title and abstract screening identified 50 eligible studies. The remaining 50 studies underwent full-text review, and 39 studies were subsequently excluded due to not directly comparing adenomyosis lesions and eutopic endometrium (n = 21), having no measurement of hormone receptor expression (n = 7), participants receiving hormonal treatment (n = 1), or being a review article or abstract only available (n = 7) or because we were not able to translate it into English language (n = 3). The remaining 11 studies were included. Two further studies were identified through forward and backward chaining, one of which could not be retrieved, and the other was included, resulting in a total of 12 studies included in the systematic review and thematic synthesis (Fig. 2).

### Study characteristics

There were 12 studies in total, of which 11 employed proteomic techniques and one used single-cell transcriptomics to investigate the hormone receptor expression in eutopic and ectopic endometrium in adenomyosis. Eleven studies investigated ER expression, 10 studies investigated PR expression, one study investigated AR expression, and one study investigated GnRH receptor expression (Table 1). A range of menstrual cycle stages was included. Publication dates ranged from 1979 to 2023.

**Table 1.** Summary of characteristics and main findings of the included studies.

Author, year	n	Method	Cycle phase	Hormone receptor	Endometrial subregion studied	Significant findings on the expression of hormone receptors in adenomyosis lesions compared to eutopic endometrium
Khan *et al.*, 2016	17	IHC	MP, PP, SP	ER, PR	Functionalis and basalis	ER: No significant differences PR: A significant decrease in adenomyosis lesions vs. endometrial functionalis in SP. No significant differences in MP or PP
Konopka *et al.*, 1998	37	Radioimmunoassay	PP, SP, MP	nER cER cPR	Not specified	cER: No difference in PP, possibly decreased adenomyosis lesions vs. eutopic endometrium in SP, but statistical significance was not reported nER: Increased in adenomyosis lesions vs. eutopic endometrium in PP and SP; significance not reported cPR: Increased in adenomyosis lesions vs. eutopic endometrium in PP and SP; significance not reported
Li *et al.*, 2021	120	IHC	Not specified	ERα, ERβ, GnRH-R	Not specified	ERα: No significant differences ERβ: No significant differences GnRH-R: No significant differences
Mehasseb *et al.*, 2011	54	IHC	MP, PP, SP	ERα, ERβ, PR-A, PR-B	Functionalis and basalis	ERα: A significant increase in adenomyosis lesions vs. endometrial functionalis (epithelium and stroma) in the SP ERβ: No significant differences PR-A: No significant differences PR-B: No significant differences
Nie *et al.*, 2009	50	IHC	PP, SP	PR-B	Not specified	PR-B: No significant differences
Samartzis *et al.*, 2023	73	IHC	PP, SP	GPER, ERα, ERβ, PR	Not specified	GPER: A significant decrease in adenomyosis lesions vs. eutopic endometrium (epithelium) ERα: No significant differences ERβ: No significant differences PR: No significant differences
Tamaya *et al.*, 1979	10	Radioimmunoassay	PP, SP	ER, PR, AR	Not specified	ER: No significant differences PR: No significant differences AR: No significant differences
Ueki *et al.*, 2004	78	IHC	MP, PP, SP	ER, PR	Not specified	ER: Increased in adenomyosis lesions vs. eutopic endometrium in SP. No differences in PP or MP (statistical significance not reported) PR: Decreased in adenomyosis lesions vs. eutopic endometrium. No difference in PP or MP (statistical significance not reported)
Yildiz *et al.*, 2023	3	Single-cell transcriptomics	PP	*ESR1*, *ESR2*, *PGR*	Functionalis and basalis	*ESR1* and *ESR2*: A significant increase in adenomyosis lesions vs. eutopic endometrium *PGR*: A significant increase in expression in adenomyosis lesions vs. eutopic endometrium
Zeng *et al.*, 2017	43	IHC	Not specified	ER	Not specified	ER: A significant increase in adenomyosis lesions vs. eutopic endometrium
Zhang *et al.*, 2008	46	IHC	PP	ER, PR	Not specified	ER: A significant decrease in adenomyosis lesions vs. eutopic endometrium PR: A significant decrease in adenomyosis lesions vs. eutopic endometrium
Zhang *et al.*, 1999	78	IHC	Not specified	ER, PR	Not specified	ER: A significant decrease in adenomyosis lesions vs. eutopic endometrium PR: A significant decrease in adenomyosis lesions vs. eutopic endometrium

AR, androgen receptor; cER, cytosolic oestrogen receptor; cPR, cytosolic progesterone receptor; ERα, oestrogen receptor; ERβ, oestrogen receptor beta; GnRH-R, GnRH receptor; GPER, G protein-coupled oestrogen receptor; IHC, immunohistochemistry; MP, menstrual phase; nER, nuclear oestrogen receptor; PP, proliferative phase; PR, progesterone receptor; SP, secretory phase.

### Bias and quality assessments

A formal methodological quality assessment was performed using the NOS. All studies were susceptible to selection bias. Six of the 11 studies accounted for the menstrual cycle phase in the reported results (Konopka *et al.*, 1998; Ueki *et al.*, 2004; Zhang *et al.*, 2008; Mehasseb *et al.*, 2011; Khan *et al.*, 2016; Yildiz *et al.*, 2023). Only five studies described controlling for the presence of other gynaecological co-morbidities such as uterine fibroids or endometriosis (Konopka *et al.*, 1998; Nie *et al.*, 2009; Mehasseb *et al.*, 2011; Khan *et al.*, 2016; Li *et al.*, 2021), suggesting a high risk of confounding bias. A summary of NOS scores is presented in Table 2.

**Table 2.** Summary of Newcastle–Ottawa Scale scores of included studies.

Author, year	Selection				Comparability		Exposure			Total	Quality
	1	2	3	4	5	6	7	8	9		
Khan *et al.*, 2016	*	*	*	*	*	*	*	*	*	9	Good
Konopka *et al*., 1998	*	*	*	*	*	–	*	*	*	8	Fair
Li *et al*., 2021	*	*	*	*	–	*	*	*	*	8	Fair
Mehasseb *et al*., 2011	*	*	*	*	*	*	*	*	*	9	Good
Nie *et al*., 2009	–	*	*	*	*	–	*	*	*	7	Fair
Samartzis *et al*., 2023	*	*	*	*	*	–	*	*	*	8	Fair
Tamaya *et al*., 1979	–	*	*	–	–	–	*	*	*	5	Poor
Ueki *et al*., 2004	*	–	*	–	*	–	*	*	*	6	Fair
Zeng *et al*., 2017	*	–	*	*	–	–	*	*	*	6	Poor
Zhang *et al*., 1999	*	*	*	*	–	–	*	*	*	7	Poor
Zhang *et al*., 2008	*	*	*	*	*	–	*	*	*	9	Good
Yildiz *et al*., 2023	*	*	*	*	**	*	*	*	*	9	Good

Asterisks indicate the number of points scored in each category (one asterisk, 1 point; two asterisks, 2 points). Dashes indicate where no points were scored.

### Oestrogen receptor (ER)

Eleven studies investigated the expression of ER in ectopic compared with eutopic endometrium from women with adenomyosis. Ten studies examined the protein expression of ER, of which three considered ERα, three considered ERβ, and one considered GPER. Seven studies didn’t specify the ER isoform under study. One study investigated the gene expression of *ESR1* and *ESR2* using single-cell transcriptomics.

#### *ESR1*/*ESR2*

The expression of both *ESR1* and *ESR2* was increased in adenomyosis lesions compared to matched eutopic endometrium in the PP, using single-cell RNA sequencing (sc-RNAseq, Yildiz *et al.*, 2023). Although the sample size was small (n = 3), this is the only study reporting transcriptional differences in hormone receptors in matched ectopic and eutopic endometrium in adenomyosis.

#### ER alpha (ERα)

A significant increase in ERα protein expression was observed in the epithelium and stroma of adenomyosis lesions compared to the matched endometrial functionalis endometrium in the mid-SP (Mehasseb *et al.*, 2011). This study notably considered the endometrial subregion (functionalis or basalis) and separately evaluated epithelial and stromal immunoexpression of ERα. While a trend of increasing ERα immunoexpression in the adenomyosis lesions compared to the matched endometrial basalis was noted, statistical significance was not reached. No cyclical variations in ERα immunoexpression were detected in adenomyosis lesions. Patients with coexisting endometriosis and fibroids were excluded, making these findings specific to adenomyosis endometrium.

In contrast, no significant differences in ERα protein expression were reported between adenomyosis lesions and eutopic endometrium in another study (Samartzis *et al.*, 2023). Although cyclical differences in ERα expression were noted between PP and SP in both eutopic and ectopic endometrium, samples from different phases were grouped for comparison, compromising the validity of the findings. The lack of specification regarding the endometrial subregion examined and the use of a tissue microarray with only two 0.6 mm diameter cores from each region limited the ability of their data to allow accurate conclusions on hormone receptor expression. Similarly, no significant differences in ERα protein expression between ectopic and eutopic endometrium from women with adenomyosis were reported by Li *et al.* (2021). However, the menstrual cycle phase of included samples was not reported, which introduces a high level of bias as ERα exhibits cyclical variation in the endometrium.

#### ER beta (ERβ)

No significant differences in ERβ immunoexpression were observed in the epithelial or stromal cells of adenomyosis lesions compared with the matched endometrial functionalis and basalis in a well-designed study by Mehasseb *et al.* (2011). Consistent with these findings, Samartzis *et al.* (2023) and Li *et al.* (2021) also reported no significant differences in ERβ protein expression in their cohorts, although the studies had a higher chance of bias due to lack of consideration of the menstrual cycle phase or endometrial subregion.

#### G protein-coupled oestrogen receptor (GPER)

One study investigated the immunoexpression of GPER in adenomyosis lesions compared to matched eutopic endometrium (Samartzis *et al.*, 2023). GPER immunoexpression was notably higher in stromal cells compared to epithelial cells, particularly showing a more pronounced difference in eutopic endometrium compared with adenomyosis lesions. There was a significant decrease in GPER expression between the PP and SP within the eutopic endometrium, a pattern not observed in adenomyosis lesions. There was a significant decrease in epithelial expression of GPER in adenomyosis lesions compared to eutopic endometrium, although the endometrial subregions were not considered separately. This decreased GPER expression in the epithelium of adenomyosis lesions could influence the pathogenesis of adenomyosis and its response to hormonal treatments, as GPER is known to mediate rapid non-genomic oestrogen signalling, which can affect cell proliferation and migration.

#### Studies not differentiating between ER isoforms

Seven studies investigated ER protein expression in adenomyosis lesions compared to the eutopic endometrium, without differentiation of the isoform (Tamaya *et al.*, 1979; Konopka *et al.*, 1998; Zhang *et al.*, 1999; Ueki *et al.*, 2004; Zhang *et al.*, 2008; Khan *et al.*, 2016; Zeng *et al.*, 2017).

Ueki *et al.* (2004) reported an increase in ER protein expression in adenomyosis lesions evident in the SP but not in the mid-proliferative or late PP. Konopka *et al.* (1998) also observed increased concentrations of nuclear ER (ERn) in adenomyosis lesions in the SP, as well as the PP and menstrual phases, although statistical significance was not explicitly stated, and the sample size was small. When the menstrual cycle phase was not considered, an increase in ER protein expression was noted in adenomyosis lesions compared to the eutopic endometrium (Zeng *et al.*, 2017).

Tamaya *et al.* (1979) found no significant difference in ER expression between adenomyotic lesions and corresponding eutopic endometrium. This study had a small sample size (n = 10) and grouped menstrual cycle phases in their analysis, limiting the study’s power. One study considered the endometrial subregions in their analysis (Khan *et al.*, 2016) and found no significant differences in ER immunoexpression in adenomyosis lesions compared to the functionalis or basalis in all menstrual cycle phases. However, the method of confirming the menstrual cycle phase was not reported.

In contrast, Zhang *et al.* (1999) and Zhang *et al.* (2008) reported a significant decrease in immunoexpression of ER in adenomyosis lesions compared to the eutopic endometrium. Zhang *et al.* (1999) did not specify the menstrual phase cycle, while Zhang *et al.* (2008) included only PP samples. Neither study considered the endometrial subregions or specified the epithelial or stromal immunostaining comparisons.

It is important to acknowledge that the expression of ER in the endometrium exhibits significant fluctuations throughout the menstrual cycle, particularly within the hormonally responsive endometrial functionalis layer, reaching peak levels during the E2-dominant PP. Accurate assessment of these cyclical variations requires consideration of the endometrial subregions. However, this aspect was not considered in the majority of the studies reviewed here, with only two exceptions (Mehasseb *et al.*, 2011; Khan *et al.*, 2016).

### Progesterone receptor (PR)

Ten studies investigated the expression of PR in adenomyosis lesions compared with eutopic endometrium. Nine studies investigated protein expression, of which one considered PR-A (Mehasseb *et al.*, 2011) and two considered PR-B (Nie *et al.*, 2009; Mehasseb *et al.*, 2011). The remaining seven studies did not specify the PR isoform being studied (Tamaya *et al.*, 1979; Konopka *et al.*, 1998; Ueki *et al.*, 2004; Zhang *et al.*, 2008; Khan *et al.*, 2016; Samartzis *et al.*, 2023). One study only investigated the gene expression of *PGR* in PP samples (Yildiz *et al.*, 2023).

#### *PGR* gene expression

One study employing sc-RNAseq reported an increase in *PGR* expression in adenomyosis lesions compared to the matched PP eutopic endometrium (Yildiz *et al.*, 2023).

#### PR-A protein expression

Mehasseb *et al.* observed no significant differences in PR-A expression in the epithelium or stroma of adenomyosis lesions compared to the matched eutopic endometrial functionalis or basalis layers across the menstrual cycle (Mehasseb *et al.*, 2011).

#### PR-B protein expression

Two studies investigated PR-B protein expression (Nie *et al.*, 2009; Mehasseb *et al.*, 2011). Nie *et al.* (2009) found no significant difference in PR-B expression between ectopic and eutopic endometrium in adenomyosis. A similar study by Mehasseb *et al.* (2011) also reported no differences in PR-B expression between the adenomyosis lesions and both subregions of the eutopic endometrium across the menstrual cycle.

#### Studies not differentiating between PR isoforms

Seven studies did not specify the PR isoforms being studied (Tamaya *et al.*, 1979; Konopka *et al.*, 1998; Zhang *et al.*, 1999; Ueki *et al.*, 2004; Zhang *et al.*, 2008; Khan *et al.*, 2016; Samartzis *et al.*, 2023). In most studies, the endometrial subregion was not indicated, and epithelial or stromal cell types were not considered separately, except in studies by Khan *et al.* (2016) and Samartzis *et al.* (2023), respectively.

Konopka *et al.* observed increased expression of cytoplasmic PR (cPR) but no differences in nuclear PR (nPR) in adenomyotic lesions compared to the eutopic endometrium in a small number of samples from the PP and SP (Konopka *et al.*, 1998). However, statistical significance was not reported, possibly due to a small sample size (n = 37).

Ueki *et al.* (2004) observed decreased immunoexpression of PR in the adenomyotic lesions compared to the eutopic endometrium in the SP but no difference in the proliferative or menstrual phases. Notably, statistical testing of the findings was not reported. Similarly, Zhang *et al.* (1999) and Zhang *et al.* (2008) observed significantly lower PR immunoexpression in adenomyotic lesions compared to eutopic endometrium. Zhang *et al.* (1999) did not specify the menstrual cycle phase, whereas Zhang *et al.* (2008) reported PP samples only. Khan *et al.* (2016) reported significantly lower PR immunoexpression in adenomyosis lesions compared to the matched endometrial basalis in the SP. PR was described as being immunolocalized to both epithelial and stromal cells; however, the authors did not specify which cell type contributed to the immunoscores that were reported in the study. The menstrual cycle phase of the participants was reported as mostly the SP; however, three participants were noted to have irregular menstrual cycles, and the method of confirming the menstrual cycle phase was not specified. No significant difference in the expression of PR in the adenomyotic lesions and the corresponding eutopic endometrium was reported in two studies (Tamaya *et al.*, 1979; Samartzis *et al.*, 2023). Both studies considered PP and SP samples. Tamaya *et al.* conducted a radioimmunoassay study on a small sample size (n = 10), whereas Samartzis *et al.* used a larger sample size (n = 73) and investigated PR expression using immunohistochemistry (IHC).

### Androgen receptor

One study investigated the protein expression of AR in adenomyosis lesions compared with eutopic endometrium from 10 patients (Tamaya *et al.*, 1979). No significant differences in the immunoexpression of AR in the adenomyotic lesions and the corresponding eutopic endometrium in the PP or SP were observed, without consideration of the endometrial subregions.

### GnRH receptor

One study investigated the protein expression of GnRH receptor (GnRH-R) in adenomyosis lesions compared with matched eutopic endometrium from 60 patients (Li *et al.*, 2021). No significant differences were observed in GnRH-R immunoexpression between the adenomyosis lesions and eutopic endometrium. However, the menstrual cycle phase of the included samples was not reported.

## Discussion

**Figure 3.**
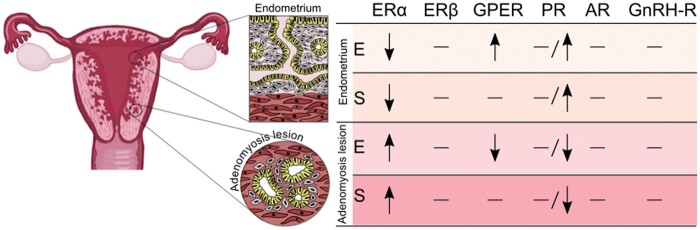
**Schematic representation summarizing the hormone receptor expression in adenomyosis lesions compared to the eutopic endometrium from the adenomyosis uteri from the 12 articles included in the systematic review.** Arrows indicate increased or decreased levels, and dashes indicate no differences. ERα, oestrogen receptor alpha; ERβ, oestrogen receptor beta; GPER, G protein-coupled oestrogen receptor; PR, progesterone receptor; AR, androgen receptor; GnRH-R, GnRH receptor; E, epithelium; S, stroma.

This systematic review summarizes the available evidence on the expression of hormone receptors in adenomyosis lesions compared to the eutopic endometrium in women with adenomyosis. A schematic representation of the main findings can be seen in Fig. 3. The current literature on hormone receptor expression in adenomyosis lesions and eutopic endometrium is constrained by technical variations and insufficient consideration of known differences that are related to menstrual cycle phases or endometrial subregions. Existing high-quality studies indicate elevated ER levels in adenomyosis lesions, inconclusive findings regarding PR levels, and a notable research gap concerning AR expression in adenomyosis lesions. Future investigations should minimize bias through well-defined cohorts, leading to robust exploration of hormone receptor profiles in adenomyosis lesions to unveil therapeutic targets and deepen our understanding of adenomyosis pathogenesis.

### Secretory-phase-specific increased ERα in adenomyosis lesions suggests hyper-oestrogenism and possible progesterone resistance

The consistent increase in ER protein expression in adenomyosis lesions (Zhang *et al.*, 1999; Zeng *et al.*, 2017), particularly during the SP (Konopka *et al.*, 1998; Ueki *et al.*, 2004; Mehasseb *et al.*, 2011), provides evidence for hyper-oestrogenism to play a role in the pathogenesis of adenomyosis. This pattern suggests a disruption of normal hormonal regulation of the endometrium-like cells in these lesions, potentially contributing to their ectopic growth and persistence. The increased expression of *ESR1* and *ESR2* in PP adenomyosis lesions, although based on limited data, may indicate a heightened sensitivity to E2 in these tissues.

In the correctly located eutopic endometrium, E2 exposure during the PP results in the upregulation of ERα, particularly in the hormonally responsive endometrial functionalis but also in the basalis (Critchley and Saunders, 2009). Progesterone action in the SP opposes the mitogenic effects of E2 in the endometrium, demonstrated by reduced expression of ERα and PR, reduced proliferation, and promotion of differentiation and decidualization of functionalis, a hallmark of endometrial receptivity (Snijders *et al.*, 1992; Critchley *et al.*, 2001; Hapangama, 2003). The endometrial basalis demonstrates less hormonal responsiveness in the SP with continued higher expression levels of ERα and PR, differentiating the two regions regarding their functional capacity.

The observation that increased ERα immunoexpression in adenomyosis lesions is more pronounced when compared to the endometrial functionalis rather than the basalis (Mehasseb *et al.*, 2011) supports the theory that adenomyosis lesions originate from invading basalis cells (Leyendecker and Wildt, 2011). In addition to providing insight into the cellular origin of adenomyosis, this finding may have implications for targeted therapies in future. ERβ immunoexpression showed no significant differences between adenomyosis lesions and matched eutopic endometrium in all studies included in this review, whether the menstrual cycle phase and endometrial subregion were considered (Mehasseb *et al.*, 2011) or not (Li *et al.*, 2021; Samartzis *et al.*, 2023). In cycling endometrium, ERβ is considered to play a role in counteracting the mitogenic effects of E2 via ERα (Hapangama *et al.*, 2015), and as such, displays lower levels in the SP endometrial functionalis than the basalis (Lecce *et al.*, 2001; Mylonas *et al.*, 2004; Critchley and Saunders, 2009). The uniformity in ERβ immunoexpression levels between adenomyosis lesions and the eutopic endometrium suggests stability in its expression between the two regions. We hypothesize that the proposed increase in local E2 levels in adenomyosis may primarily bind to ERα and induce its own expression. However, with only one study demonstrating a specific comparison of ERβ immunoexpression in adenomyosis lesions to the eutopic endometrial subregions, it is not possible to make reliable conclusions regarding the role of ERβ in adenomyosis, and thus, future studies are needed.

The collective evidence points towards increased ER expression, likely ERα, in adenomyosis lesions compared to eutopic endometrium, particularly in the SP. In the absence of cyclical variation in ER expression in adenomyosis lesions (Mehasseb *et al.*, 2011), this suggests increased exposure to oestrogen within these lesions, and ERα could mediate the trophic effect of E2 within the lesions. Prolonged and increased ER expression may result in producing an oestrogen-dominant status and a functional progesterone resistance (Vannuccini *et al.*, 2017). However, the heterogeneity and methodological limitations of existing studies necessitate further research to elucidate the precise role of ERα in the proliferation and survival of adenomyosis lesions within the myometrium and its association with progesterone resistance.

### GPER

GPER mediates the non-genomic and more immediate effects of oestrogens within the endometrium, including secondary messenger mobilization, e.g. cAMP (Gu and Moss, 1996; Revankar *et al.*, 2005). Its expression is known to fluctuate throughout the menstrual cycle, peaking in the PP, indicating its regulation by E2 (Kolkova *et al.*, 2010), and decreasing in the SP, under the influence of progesterone via PR (Kolkova *et al.*, 2010; Plante *et al.*, 2012).

One study reported a significant decrease in GPER immunoexpression in the epithelium of adenomyosis lesions compared to the eutopic endometrium (Samartzis *et al.*, 2023). However, this finding should be interpreted cautiously due to methodological limitations. The study grouped samples from different menstrual cycle phases for analysis, potentially obscuring the cyclical variations in GPER expression. Interestingly, other studies have shown increased GPER expression in both epithelial and stromal cells of eutopic endometrium from patients with adenomyosis and endometriosis compared to healthy endometrium (Plante *et al.*, 2012; Samartzis *et al.*, 2012, 2023).

The increased GPER expression observed in stromal cells of adenomyosis eutopic endometrium may potentially extend to the stromal cells of adenomyosis lesions, although this has not been explicitly reported in the literature. If such an increase does occur in both regions, it could explain the lack of significant differences in GPER immunoexpression between stromal cells of adenomyosis lesions and eutopic endometrium reported by Samartzis *et al.* (2012, 2023). Given the role of GPER in regulating cellular survival and growth (Jacenik *et al.*, 2016) and its association with fibroblast migration in other tissues (Madeo and Maggiolini, 2010), further studies are necessary to elucidate the role of GPER in adenomyosis lesion growth and survival.

### Progesterone receptor

In the endometrium, PR is upregulated by E2 via ERα in the PP in both epithelial and stromal cells of the endometrial functionalis and basalis. Conversely, progesterone downregulates PR and ERα in the SP, which is more evident in the hormonally responsive endometrial functionalis. It is generally accepted that the response to progesterone is determined by the combined actions of PR-A and PR-B (Patel *et al.*, 2015). Both PR-A and PR-B isoforms are present in the endometrial epithelium during the PP, and in the late SP, PR-A levels decline. In contrast, PR-B remains constant in the epithelial cells, suggesting that it is involved in the control of glandular secretions. In endometrial stromal cells, PR-A expression is predominant across the menstrual cycle (Mote *et al.*, 1999). In the presence of progesterone ligand, PR (especially PR-B) levels are inversely related to the transcriptional activity of *PGR* (Shen *et al.*, 2001).

Historically, technical challenges have hindered the precise assessment of PR-A and PR-B abundance and localization due to their shared sequence. The lack of specific antibodies for PR-A posed limitations in distinguishing between isoforms. However, recent advancements have led to validated antibodies capable of detecting either PR-A or PR-B isoforms (Fabris *et al.*, 2017). This allows the investigation of the distribution of PR isoforms using commercially available antibodies. It is essential to note that antibodies lacking specificity will detect both PR-A and PR-B.

The concept of progesterone resistance has emerged as a potential contributor to the pathogenesis of adenomyosis, as evidenced by the limited efficacy of progestogenic treatments in women with this condition (Moghissi, 1988; Wood, 1998). Similarly, in endometriosis, another oestrogen-driven gynaecological disorder, there seems to be a diminished responsiveness to progesterone in endometrial stromal cells, possibly linked to reduced PR expression (Bulun *et al.*, 2006). However, the conflicting results in PR expression studies highlight the complexity of this relationship and the need for more nuanced research approaches.

The majority of studies in this review reported no significant differences (Tamaya *et al.*, 1979; Zhang *et al.*, 2008; Nie *et al.*, 2009; Mehasseb *et al.*, 2011; Samartzis *et al.*, 2023) or reduced PR immunoexpression in adenomyosis lesions compared with matched eutopic endometrium (Zhang *et al.*, 1999; Ueki *et al.*, 2004; Khan *et al.*, 2016). Many of the studies included did not specify the endometrial subregion under study, nor were menstrual cycle phases specified or grouped for analysis.

The observation of reduced cytoplasmic PR in adenomyosis lesions by Konopka *et al.* (1998) is intriguing and may indicate alterations in non-genomic progesterone action, which could affect rapid cellular responses to hormonal changes. While nuclear PRs function as ligand-activated transcription factors influencing gene expression, cytoplasmic PRs are involved in non-genomic actions of progesterone (Welter *et al.*, 2003; Patel *et al.*, 2015).

The findings of Mehasseb *et al.* (2011), showing a non-significant increase in PR-A in adenomyosis lesion epithelium and, to a lesser extent, stroma, compared to the endometrial functionalis in the SP, contrast with the general trend, yet the observed discrepancy underscores the importance of considering both PR isoforms separately and examining different endometrial subregions. It is possible that adenomyosis lesions exhibit dysregulation of PR expression compared to the eutopic endometrium. However, the link with progesterone resistance cannot be concluded from the heterogeneous studies in this review. PR expression is downregulated by exposure to progesterone in the SP functionalis; therefore, reduced immunoexpression of PR can be considered a normal response to progesterone exposure. Conversely, in the PP, the upregulation of PR is mediated by E2 acting via ERα. Therefore, lower expression of PR in this phase may indicate altered oestrogen exposure, as shown by Zhang *et al.* (2008). The endometrial basalis exhibits less hormone responsiveness, exemplified by higher PR expression than the functionalis in the SP. In this regard, adenomyosis lesions are dissimilar to the basalis endometrium, demonstrating lower PR expression in the SP, which may indicate progesterone responsiveness. Given the known cyclical and regional variations in PR expression in the endometrium being indicative of its function, it is essential for future studies to factor this into experimental designs. Notably, the comparison to the endometrial functionalis is essential.

### Androgen receptor

While the evidence regarding AR expression in adenomyosis lesions is limited, the findings from Tamaya *et al.* (1979) suggest a potential decrease in AR in these lesions compared to the eutopic endometrium. Although not statistically significant, this trend warrants further investigation. The precise role of androgens acting through AR in the human endometrium, which is exposed to high concentrations of oestrogen and progesterone, is complex and not fully understood. Nevertheless, research has shown that AR is more prevalent in the functionalis stroma during the PP compared to the SP (Mertens *et al.*, 2001; Slayden *et al.*, 2001; Slayden and Brenner, 2004; Critchley and Saunders, 2009; Kamal *et al.*, 2016), and in contrast, in the epithelium, AR is associated with proliferative quiescence (Kamal *et al.*, 2016; Maclean *et al.*, 2020). This observation hints at a potential involvement of AR in the regulation of cellular proliferation processes. In the context of adenomyosis, altered AR expression could contribute to the pathogenesis, as AR can interact with both oestrogen and progesterone signalling pathways. Altered AR expression could therefore indirectly affect the response of the tissue to these hormones, potentially contributing to the oestrogen dominance.

Future studies are imperative to define the exact role of AR in adenomyosis lesions comprehensively. Investigating the interplay between androgens, AR, and other hormonal factors within the context of adenomyosis will provide valuable insights into the pathogenesis and potential therapeutic targets for this condition.

### GnRH receptor

In the context of the endometrium, GnRH receptors (GnRH-R) have been identified in both epithelial and stromal cells, with their expression peaking during the SP (Raga *et al.*, 1998). GnRH agonists can enhance human endometrial stromal cell motility by activating matrix metalloproteinases (MMPs), suggesting a role for locally expressed GnRH in regulating extracellular matrix degradation (Wu *et al.*, 2015). The presence of GnRH-R is evident in limited studies of eutopic and ectopic endometrium in adenomyosis (Li *et al.*, 2021), and treatment with GnRH analogues has shown efficacy in managing adenomyosis-associated symptoms such as pain and abnormal bleeding (Ishihara *et al.*, 2003). Evidence in endometriosis has indicated that GnRH analogues may directly affect peripheral GnRH-R and pituitary function (Borroni *et al.*, 2000; Khan *et al.*, 2010).

While the study by Li *et al.* (2021) found no difference in GnRH-R immunoexpression between adenomyosis lesions and eutopic endometrium, the lack of menstrual cycle phase specification limited the interpretation of these findings. The cyclical variation of GnRH-R expression in normal endometrium, with peak expression during the SP, indicates a complex regulatory mechanism that may be disrupted in adenomyosis. This disruption could contribute to the abnormal growth and invasion of endometrial tissue into the myometrium, which is characteristic of adenomyosis. Consequently, further investigations are warranted to delve deeper into the expression patterns of GnRH-R in adenomyosis lesions to enhance our understanding of their role.

### Direction for future research

The future of research in hormone receptor expression in adenomyosis should focus on comprehensive, multi-faceted approaches that address the current limitations in our understanding. Priority should be given to studies that simultaneously examine multiple hormone receptors (ER, PR, AR, GnRH-R, GPER) across different menstrual cycle phases and compare the adenomyosis lesions to specific endometrial subregions. The integration of advanced techniques such as single-cell and spatial transcriptomics and epigenetic profiling could provide insights into cell-specific receptor dynamics and regulatory mechanisms. Longitudinal studies correlating receptor profiles with disease progression, associated symptoms, and response to available treatments will be crucial for developing personalized therapeutic strategies. Furthermore, functional studies using *in vitro* and *in vivo* models should investigate the interplay between different hormonal pathways and their roles in adenomyosis pathogenesis. By combining these approaches with genetic analyses and exploration of non-genomic hormone actions, future research has the potential to unravel the complex hormonal regulation of adenomyosis, leading to the development of novel, targeted therapies that can effectively modulate the unique receptor landscape of adenomyosis lesions and improve patient outcomes.

### Strengths and limitations

This review is strengthened by the use of the systematic methodology within PRISMA guidelines, the use of the Newcastle–Ottawa scoring system to evaluate the level of bias from each study, and the consideration of the impact of confounding factors such as menstrual cycle phase and endometrial subregion.

This review has limitations, primarily stemming from the heterogeneity of the included studies. There was a high level of bias in some included studies due to samples from different menstrual cycle phases being grouped for analysis and the coexistence of gynaecological morbidities such as endometriosis, which may affect the hormone receptor profile of the eutopic endometrium. In terms of laboratory techniques employed, IHC allows only semi-quantitative immunostaining analysis. However, IHC is useful as it allows for consideration of region-specific differences in the endometrium, as well as consideration of the epithelial and stromal cell immunoexpression. Most studies employing IHC did not comment on region-specific differences in the endometrium. Given the well-documented cyclical variations in hormone receptor expression within the endometrium, the need for more attention to region-specific differences represents a notable limitation in the current body of literature.

Only two studies delineated the adenomyosis subtype (diffuse or focal) among participants, yet neither delved into how these subtypes might influence hormone receptor expression. Emerging evidence suggests distinct pathogeneses for different adenomyosis subtype lesions (Brosens *et al*., 2015; Chapron *et al*., 2017); future investigations should explore potential associations between adenomyosis subtype and hormone receptor profiles. However, this is currently impeded by the lack of standardized criteria for diagnosing adenomyosis subtypes. If there are adenomyosis subtype-specific differences in hormone receptor expression, this may be relevant to lesion-specific symptoms and responses to treatments.

Studying the transcriptome of adenomyosis lesions is challenging with most traditional transcriptional techniques, such as PCR, due to the difficulties in isolating adenomyotic tissue from the myometrium. However, recent advancements in single-cell sequencing, as exemplified by Yildiz *et al.* (2023), and spatial transcriptomic technologies provide the opportunity to investigate the gene expression of adenomyosis lesions compared to the endometrial subregions. Future research should consider complicated aspects of the human endometrium (region-specific differences) and adenomyotic lesions (e.g. subtype-specific changes) and use the novel innovative methods to unravel the intricate molecular landscape of hormone receptor expression in adenomyosis lesions.

## Conclusion

This systematic review provides the first comprehensive analysis of hormone receptor expression in adenomyosis lesions compared to matched endometrium, considering endometrial subregions and epithelial or stromal cell types. The findings provide insights into the pathogenesis of adenomyosis. The review demonstrates consistent evidence of increased ER levels in adenomyosis lesions, particularly during the SP, supporting the hypothesis of hyper-oestrogenism in adenomyosis lesions, which may drive their growth and persistence even in the presence of circulating progesterone. Interestingly, our analysis of PR expression revealed a more nuanced picture than previously thought. While some studies suggest reduced PR levels in adenomyosis lesions, the overall evidence does not support a clear-cut progesterone resistance mechanism. Instead, the available data point towards a complex dysregulation of progesterone signalling, which may vary depending on the specific PR isoform and the endometrial subregion examined. This finding challenges the simplistic view of adenomyosis as a purely progesterone-resistant condition and suggests that some lesions may retain partial progesterone responsiveness. There is a notable research gap concerning AR levels in adenomyosis lesions, which is an area for future research given the potential role of AR in endometrial proliferation. Future research should focus on comprehensive, well-designed studies that consider menstrual cycle phases, comparison with matched specific endometrial subregions, and analysis of multiple receptor types simultaneously. Such studies will be crucial for developing targeted treatments that can effectively modulate the hormonal environment of adenomyosis lesions and improve outcomes for patients with this challenging condition.

## Supplementary Material

hoaf002_Supplementary_Data

## Data Availability

The data underlying this article will be shared on reasonable request to the corresponding author.
