# Supplementary material for: Hormone receptor profile of ectopic and eutopic endometrium in adenomyosis: a systematic review
Source: Hum Reprod Open. 2025 Jan 20;2025(1):hoaf002. doi: 10.1093/hropen/hoaf002 (PMC11810641; doi:10.1093/hropen/hoaf002)
Supplement: hoaf002_Supplementary_Data [file hoaf002_supplementary_data.zip › 25523_R2 Supplementary tables.docx]

**Supplementary Table S1:** Search strategy

| **Database** | **Date** | **Search strategy** | **Results** |
| --- | --- | --- | --- |
| Medline | 16/05/2024 | 1. Adenomyosis/ or Adenomyo* [5217] 2. estrogen receptor or ER [150,585] 3. progesterone receptor or PR [79,521] 4. androgen receptor or AR [77,651] 5. hormone receptor [30250] 6. 2 or 3 or 4 or 5 [310,627] 7. 1 and 6 [204] | 204 |
| Pubmed | 16/05/2024 | 1. Adenomyosis[MeSH Terms] OR adenomyo* [5229] 2. Estrogen receptor OR ER [229,457] 3. Progesterone receptor OR PR [554,214] 4. Androgen receptor OR AR [262,169] 5. Hormone receptor [339,554] 6. 2 OR 3 OR 4 OR 5 [1,242,058] 7. 1 AND 6 [375] | 375 |
| Scopus | 16/05/2024 | 1. TITLE (adenomyo*) [3,831] 2. ( ( ALL ( "hormone receptor" ) OR ALL ( "Estrogen receptor" ) OR ALL ( er ) OR ALL ( "Progesterone receptor" ) OR ALL ( pr ) OR ALL ( "Androgen receptor" ) OR ALL ( ar ) ) ) [3,490,335] 3. 1 and 2 [616] | 616 |
| Embase | 16/05/2024 | 1. Adenomyosis/ or Adenomyo* [10,021] 2. estrogen receptor or ER [262,019] 3. progesterone receptor or PR [486,208] 4. androgen receptor or AR [123,570] 5. hormone receptor [75054] 6. 2 or 3 or 4 or 5 [863,411] 7. 1 and 6 [710] | 710 |
| Cochrane central library | 16/05/2024 | 13 results non relevant (all clinical trials or reviews or protocols)  adenomyo* AND ('hormone receptor' OR 'estrogen receptor' OR ER OR 'progesterone receptor' OR PR OR 'androgen receptor' OR AR) | 0 |

**Supplementary Table S2:** Thematic synthesis

| **Codes** | **Relevant studies** | **Subthemes** | **Themes** |
| --- | --- | --- | --- |
| Diffuse adenomyosis | Yildiz *et al*., 2023 | Adenomyosis phenotype | Adenomyosis classification |
| Focal adenomyosis | No studies |  |  |
| Diffuse and focal adenomyosis | No studies |  |  |
| Proliferative samples only | Zhang *et al*., 2008, Yildiz *et al*., 2023 | Proliferative samples only | Menstrual cycle variation |
| Secretory samples only | No studies | Secretory samples only |  |
| Proliferative and secretory samples | Nie *et al*., 2009, Samartzis *et al*., 2023, Konopka *et al*., 1998 | Proliferative and secretory samples |  |
| Proliferative, secretory and menstrual samples | Mehasseb *et al*., 2011, Ueki *et al*., 2004 | Proliferative, secretory and menstrual samples |  |
| No difference in adenomyosis lesions compared to matched eutopic endometrium | No studies | ER (subtype not specified) | Oestrogen receptors |
| Increase in adenomyosis lesions compared to matched eutopic endometrium | Ueki *et al*., 2004, Zeng *et al*., 2017 |  |  |
| Decrease in adenomyosis lesions compared to matched eutopic endometrium | Zhang *et al*., 2008, Tamaya *et al*., 1979, Zhang *et al*., 1999, Konopka *et al*., 1998 |  |  |
| No difference in adenomyosis lesions compared to matched eutopic endometrium | Li *et al*., 2021, Samartzis *et al*., 2023 | ERα/*ESR1* |  |
| Increase in adenomyosis lesions compared to matched eutopic endometrium | Mehasseb *et al*., 2011, Yildiz *et al*., 2023 |  |  |
| Decrease in adenomyosis lesions compared to matched eutopic endometrium | No studies |  |  |
| No difference in adenomyosis lesions compared to matched eutopic endometrium | Mehasseb *et al*., 2011, Li *et al*., 2021, Samartzis *et al*., 2023 | ERβ/*ESR2* |  |
| Increase in adenomyosis lesions compared to matched eutopic endometrium | Yildiz *et al*., 2023 |  |  |
| Decrease in adenomyosis lesions compared to matched eutopic endometrium | No studies |  |  |
| No difference in adenomyosis lesions compared to matched eutopic endometrium | Samartzis *et al*., 2023 | PR (subtype not specified)/*PGR* | Progesterone receptors |
| Increase in adenomyosis lesions compared to matched eutopic endometrium | Yildiz *et al*., 2023, Konopka *et al*., 1998 |  |  |
| Decrease in adenomyosis lesions compared to matched eutopic endometrium | Nie *et al*., 2009, Zhang *et al*., 2008, Ueki *et al*., 2004, Tamaya *et al*., 1979, Zhang *et al*.,1999 |  |  |
| No difference in adenomyosis lesions compared to matched eutopic endometrium | No studies | PR-A |  |
| Increase in adenomyosis lesions compared to matched eutopic endometrium | Mehasseb *et al*., 2011 |  |  |
| Decrease in adenomyosis lesions compared to matched eutopic endometrium | No studies |  |  |
| No difference in adenomyosis lesions compared to matched eutopic endometrium | Mehasseb *et al*., 2011 | PR-B |  |
| Increase in adenomyosis lesions compared to matched eutopic endometrium | No studies |  |  |
| Decrease in adenomyosis lesions compared to matched eutopic endometrium | Nie *et al*., 2004 |  |  |
| No difference in adenomyosis lesions compared to matched eutopic endometrium | No studies | AR | Androgen receptor |
| Increase in adenomyosis lesions compared to matched eutopic endometrium | No studies |  |  |
| Decrease in adenomyosis lesions compared to matched eutopic endometrium | Tamaya *et al*., 1979 |  |  |
| No difference in adenomyosis lesions compared to matched eutopic endometrium | No studies | GPER | GPER |
| Increase in adenomyosis lesions compared to matched eutopic endometrium | No studies |  |  |
| Decrease in adenomyosis lesions compared to matched eutopic endometrium | Samartzis *et al*., 2023 |  |  |
| Increase in adenomyosis lesions compared to matched eutopic endometrium | No studies | GnRH-R | GnRH-R |
| Decrease in adenomyosis lesions compared to matched eutopic endometrium | No studies |  |  |
| No difference in adenomyosis lesions compared to matched endometrium | Li *et al*., 2021 |  |  |

AR, androgen receptor; ER, oestrogen receptor; ERα, oestrogen receptor; ERβ, oestrogen receptor beta; ESR1, oestrogen receptor 1; ESR2, oestrogen receptor 2; GnRH-R, gonadotrophin releasing hormone receptor; GPER, G protein-coupled oestrogen receptor; PGR, progesterone receptor; PR, progesterone receptor; PR-A, progesterone receptor A; PR-B, progesterone receptor B.
